# A Machine Learning‐Driven Pore‐Scale Network Model Coupling Reaction Kinetics and Interparticle Transport for Catalytic Process Design

**DOI:** 10.1002/advs.202513649

**Published:** 2025-12-03

**Authors:** Ming‐Liang Qu, Zhao‐Bin Ding, Dingyue Zhang, Sajjad Foroughi, Huanhao Chen, Zi‐Tao Yu, Jiyizhe Zhang, Lifeng Xiao, Martin J. Blunt, Xiaolei Fan, Qingyang Lin

**Affiliations:** ^1^ State Key Laboratory of Clean Energy Utilization Zhejiang University Hangzhou 310027 China; ^2^ Wenzhou Key Laboratory of Novel Optoelectronic and Nano Materials Institute of Wenzhou Zhejiang University Wenzhou 325006 China; ^3^ Department of Earth Science and Engineering Imperial College London London SW7 2AZ UK; ^4^ School of Chemical Engineering and Technology Sun Yat‐sen University Zhuhai 519028 China; ^5^ State Key Laboratory of Materials‐Oriented Chemical Engineering College of Chemical Engineering Nanjing Tech University Nanjing 210009 China; ^6^ Department of Chemical Engineering and Biotechnology University of Cambridge Cambridge CB3 0AS UK; ^7^ Department of Chemical Engineering School of Engineering The University of Manchester Manchester M13 9PL UK; ^8^ Green Synenergy Co. No. 1299 Kehai Avenue Shaoxing 312000 China; ^9^ Nottingham Ningbo China Beacons of Excellence Research and Innovation Institute University of Nottingham Ningbo China 211 Xingguang Road Ningbo 315100 China

**Keywords:** Catalytic processes, CO_2_ hydrogenation, Kinetics, Machine learning, Pore‐scale modeling, Porous media

## Abstract

A pore‐scale dual‐network model is presented with kinetics (DNMK), enhanced by machine learning (ML), for efficient multiscale modeling of reaction‐transport coupled catalytic processes in porous systems. In such systems, apparent catalytic performance arises from the intricate interplay between intrinsic microkinetics and inter‐particle transport phenomena. By explicitly resolving these coupled effects, DNMK provides mechanistic insight into how spatial particle arrangements and transport limitations govern overall reactor performance. A key innovation of this work is the integration of ML‐based surrogates to accelerate the microkinetic module, effectively bridging the large spatial and temporal scale mismatches between transport and catalytic reactions. This hybrid approach achieves up to a 750‐fold computational speed‐up while preserving full physical and chemical fidelity. The framework is demonstrated for sorption‐enhanced CO_2_ hydrogenation to methanol, where DNMK identifies optimal catalyst‐sorbent configurations that maximize apparent activity and reactor‐scale performance. More broadly, DNMK establishes a high‐resolution, ML‐driven platform for digital catalytic experimentation, enabling predictive, in silico optimization of catalyst scaling, utilization, and process intensification. By allowing rapid, physically consistent evaluation of complex catalytic systems, DNMK reduces reliance on costly experimental trials and opens new pathways for data‐driven reactor and process design across diverse chemical engineering applications.

## Graphical abstract

Designing catalytic processes in porous reactors requires resolving coupled multiscale reaction–transport phenomena. We develop a machine‐learning‐accelerated pore‐scale dual‐network model with kinetics (DNMK), which captures reaction kinetics, pore‐scale transport, and reactor‐level behavior. As a mesoscopic framework, DNMK provides microscopic insight to interpret macroscopic performance, enabling rational design of efficient and scalable heterogeneous multiphase systems.

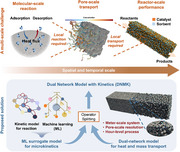

## 1 Introduction

Heterogeneous catalysis in porous media plays a central role in key chemical, environmental, and energy processes. The overall efficiency of such a system is governed by not only the intrinsic activity of the catalyst surface but also transport phenomena, i.e., the complex coupling of surface reactions with the multiscale inter‐particle transport of fluids, heat, and mass.^[^ ^1^, ^2^ ^]^ Hence, optimizing the apparent performance of a heterogeneous catalytic system in porous media requires more than designing intrinsically more active materials; it demands a mechanistic understanding of inter‐particle interactions as well, as both ultimately affect reactor‐scale behavior.^[^ ^3^, ^4^ ^]^ Compared to experimental studies, in silico optimization based on advanced models could reduce time and cost significantly, being valuable in chemical engineering designs for practical applications. This is important in the design and optimization of catalytic processes in porous systems, such as packed beds and structured foam beds, where the design and configuration of the porous network might affect transport phenomena, and thus the apparent catalytic efficiency significantly. However, modeling of such reaction‐transport coupled catalytic systems across scales remains a major challenge, as it demands accurate resolution of multiphysical processes across disparate spatial and temporal domains.

Current multi‐scale modeling approaches that couple transport phenomena with reaction kinetics often rely on homogenization assumptions and employ idealized or simplified reactor models to ensure computational feasibility. While these techniques enable multi‐scale simulations, they tend to oversimplify the inherent heterogeneity of real porous systems and microscopic details such as the dynamics of transport phenomena within the pore (void) space between the packed catalyst particles. As a result, these models lead to deviations from actual performance.^[^ ^5^, ^6^, ^7^, ^8^, ^9^, ^10^, ^11^, ^12^ ^]^ On the other hand, state‐of‐the‐art pore‐scale models, such as direct numerical simulation (DNS), can capture detailed microscopic phenomena, but their applicability is limited due to the vast differences in time and length scales between chemical reactions, heat and mass transport, and overall reactor performance. In particular, reactions and adsorption/desorption occur on micro to millisecond scales, inter‐particle transport occurs over milliseconds to seconds,^[^ ^13^, ^14^ ^]^ and reactor‐scale processes operate from seconds to hours. This timescale mismatch makes it computationally expensive to simulate reactor‐scale systems,^[^ ^11^, ^15^ ^]^ rendering current pore‐scale approaches impractical for simulating full‐scale reactors using standard computational resources.^[^ ^16^, ^17^, ^18^, ^19^ ^]^ One of the major challenges in simulating catalytic processes in porous media, therefore, lies in reconciling these disparate temporal scales between reaction kinetics, adsorption dynamics and transport phenomena. Hence, solving these processes simultaneously using conventional solvers leads to prohibitive computational costs.

Given these challenges, there is a pressing need to develop advanced models that can accurately capture both microscopic and macroscopic behavior to facilitate reactor design and optimization. An ideal model should fulfill three key criteria: i) the ability to integrate kinetic models with fluid dynamics; ii) efficient coupling and simulation of multi‐scale systems, overcoming significant timescale differences; and iii) flexibility to adapt system characteristics, such as the geometry, packing pattern and functionality of packing materials (using a packed bed reactor, PBR, as an example), for design optimization. Currently, no established method exists for accurately simulating the entire catalytic process using a pore‐scale model that encompasses a full‐sized, 3D reactor with coupled heat/mass transport and surface reactions.

**Figure 1:**
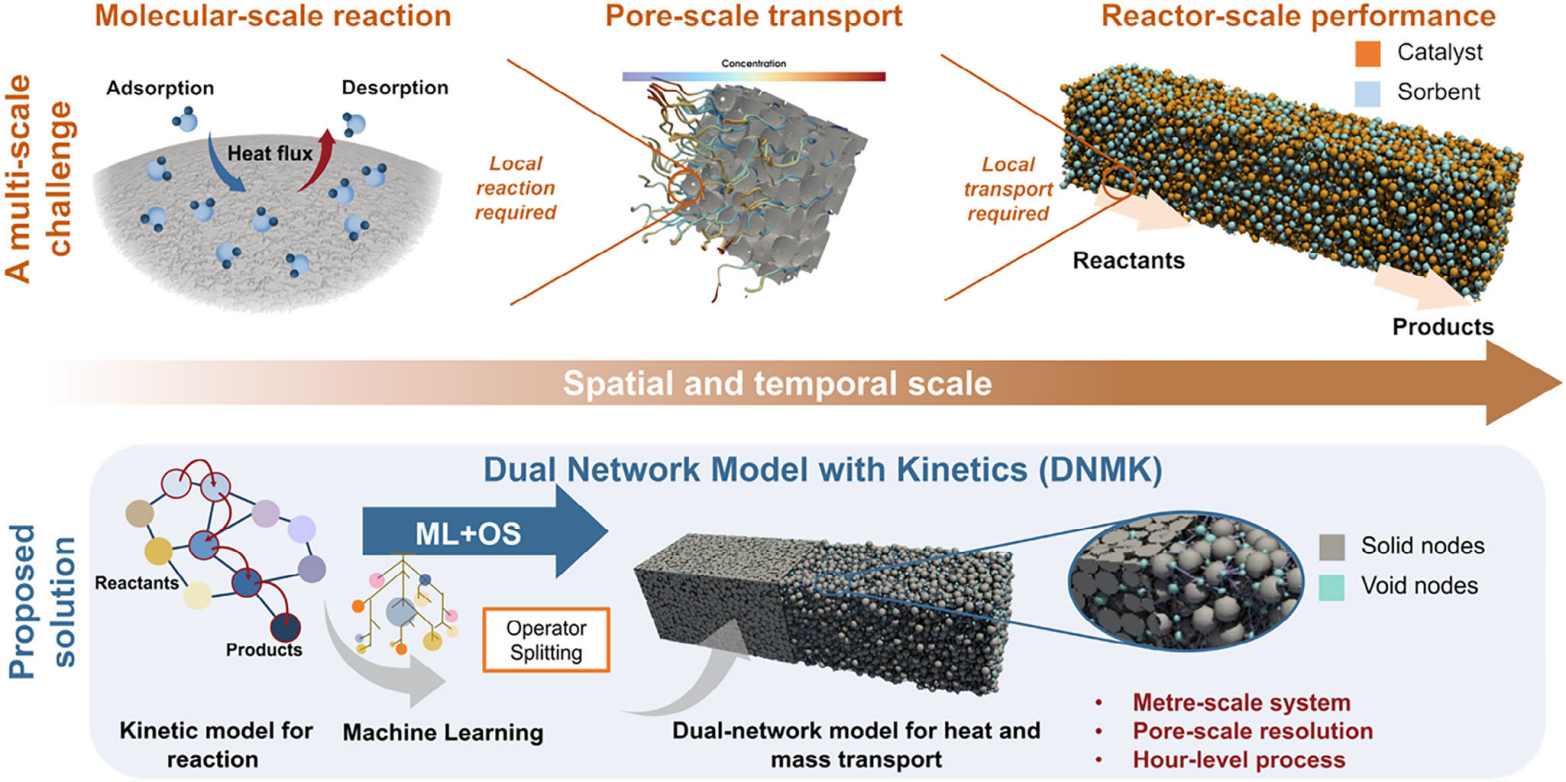
Schematic illustration of the dual network model with kinetics (DNMK) to capture the multi‐scale nature of catalytic processes in porous reactors. An operator‐splitting machine‐learning (OS‐ML) method was employed to address the mismatch in temporal resolution between the molecular and pore scales. The outcomes were then used as input parameters in the DNMK to model a full‐scale reactor over several hours.

This work proposes a novel pore‐scale dual‐network model with kinetics (DNMK) that integrates a machine learning (ML) surrogate model (Supplementary Note 1) to accelerate the simulation of coupled reaction–transport catalytic processes across multiple spatial and temporal scales (**Figure** **1**). The ML surrogate model, trained on high‐resolution kinetic data, is specifically designed to replace the conventional solver, bridging the time‐scale gap between kinetics and transport. It emulates the microkinetic processes accurately while significantly reducing computational cost. We demonstrate its capabilities through an exemplar system: a sorption‐enhanced reaction (SER) for CO_2_ hydrogenation for methanol synthesis^[^ ^20^, ^21^, ^22^, ^23^ ^]^ in a PBR consisting of catalyst and sorbent particles. The DNMK incorporates two kinetic models, namely water adsorption and CO_2_ hydrogenation, and enables each particle to be individually defined as either a sorbent or a catalyst. This allows the simulation of diverse packing configurations with varying particle distribution,^[^ ^9^, ^24^, ^25^ ^]^ facilitating detailed analysis of bed design, transport–reaction interactions, and apparent system performance. This model is validated against literature data and experimental measurements, demonstrating its significant potential as a high‐resolution, ML‐accelerated platform delivering digital experiments for catalyst scaling, utilization and process intensification in porous reactors.

## 2 Results and Discussion

### 2.1 ML‐Aided Multi‐Scale Modeling Framework with Validation

**Figure 2:**
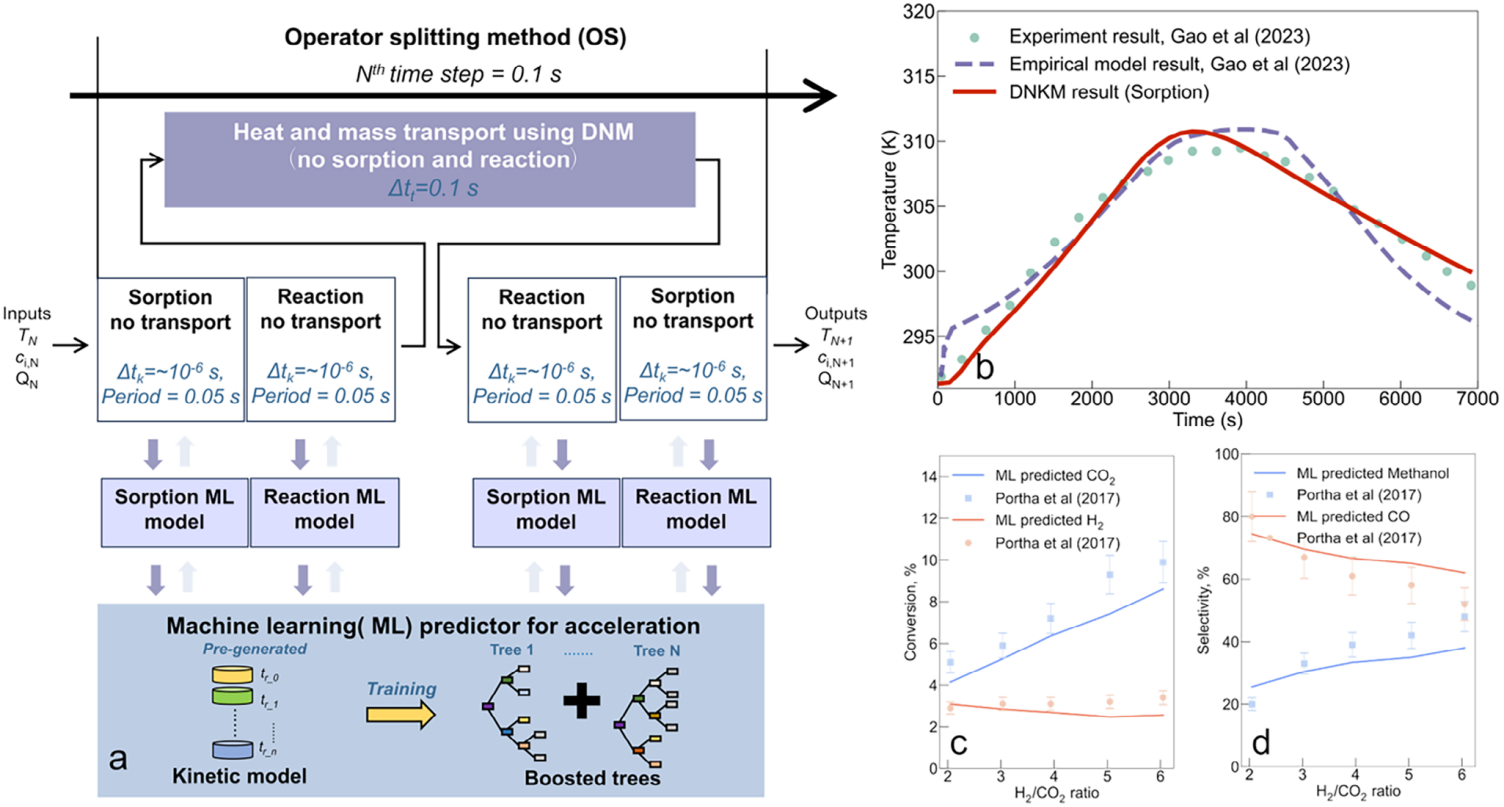
a) Schematic illustration of the OS‐ML scheme (*T* is the temperature, *c_i_* is the concentration of the *i* ^th^ species, and *Q* is the adsorption capacity). b) Validation of DNMK for water adsorption against experimental measurements and an empirical model.^[^ ^10^ ^]^ The volume‐averaged temperature at 5 cm from the inlet of a PBR as a function of time using a air‐water vapor mixture as the feed. c,d) Validation of the kinetic model for CO_2_ hydrogenation with ML against experimental data in the literature^[^ ^39^ ^]^ (error bars represent 10% a standard deviation). The prediction accuracies, reflecting the agreement between five predicted values and experimental values, are 0.85 ± 0.04 (CO_2_ conversion), 0.86 ± 0.08 (H_2_ conversion), 0.92 ± 0.06 (MeOH selectivity), and 0.85 ± 0.07 (CO selectivity).

To demonstrate the ability of the DNMK framework, we studied an example system of sorption‐enhanced CO_2_ hydrogenation to methanol in a PBR, a critical CO_2_ utilization route which involves intertwined processes including chemical reactions (the full reaction network is shown by Equations S1–S20), H_2_O sorption and pore/particle scale transport. In situ removal of water during the hydrogenation process through physical sorption can shift the equilibrium to enhance methanal productivity and overall reactor performance^[^ ^26^, ^27^, ^28^, ^29^ ^]^ through optimizing the spatial distribution of catalyst/sorbent particles. The PBR had dimensions of 10 × 2.5 × 2.5 cm^3^, which comprises 10265 particles with a diameter of 2 mm (a mixture of catalyst and sorbent particles), resulting in 11050 pores. In the DNMK, to address the time‐scale mismatch of ≈5 orders of magnitude (*Δt_k_* = ∼10^−6^ s for solving kinetic models and *Δt_t_* = ∼0.1 s for heat and mass transport), we solved heat and mass transfer equations with exothermic adsorption source terms (Equations (1), (2), (3), (4), (5), (6), (7), (8), (9), (10), (11), (12), (13)) simultaneously with sorption (a kinetic model for 13X zeolite sorbent,^[^ ^30^, ^31^ ^]^ Equation 20) and reaction (a kinetic model developed for Cu/Zn/Al_2_O_3_^[^ ^32^, ^33^ ^]^), shown in **Figure** **2**a.

To efficiently couple the DNM and kinetic models, we implemented Strang operator‐splitting (OS)^[^ ^34^ ^]^ that integrates the governing equations (Equations (10), (11), (12), (13)) every 0.1 s. This method decouples the governing equations into distinct components: heat/mass transport and source terms (change of a species concentration based on chemical reaction rates). The first step of the Strang splitting procedure is to solve the contributions from source terms through solving kinetic models (including water sorption on sorbent particles and CO_2_ hydrogenation on the catalyst particles) with 0.05 s time steps. This process updates the water saturation in the adsorbents (represented by solid nodes, *Q*, mol/kg), temperature (*T*, K), and component concentrations in the fluid phase (*c*, mol/m^3^ in the void nodes), assuming no mass and heat transport. Subsequently, the DNM solves one transport time step with *Δt_t_* = 0.1 s to update *T* and *c* without involving the kinetic models. The kinetic models are subsequently solved again for another 0.1 s as described above, and the process is repeated over the entire simulation. Detailed equations for the adsorption (Equations (14), (15), (16), (17), (18), (19), (20)), reaction kinetic model (see Supporting Information Note S2), and the Strang splitting (Equations 21 and 22) are provided in the Experimental section.

Despite the efficiency of the OS method, each 0.1 s time step involves solving stiff ordinary differential equations for sorption and reaction, leading to significant computational time for full simulation over several hours. To further accelerate the simulation, we generated 100000 datasets under various conditions using adsorption and reaction kinetic models, which were used to train a random forest (RF)‐based ML model to replicate the kinetic model that covers the reaction conditions. The RF model was selected for its efficiency in handling large, high‐dimensional datasets,^[^ ^35^ ^]^ as evidenced by the previous application to kinetic modeling studies.^[^ ^36^, ^37^, ^38^ ^]^ The ML adsorption model predicted temperature (*T*), water vapor concentration (*c_w_*), and adsorption capacity (*Q*) every 0.1 s with input ranges of 283.15–530.15 K, 0.001–1.6 mol m^−3^, and 0–17.5 mol kg^−1^ (see Figure S1 and Supplementary Note 1 for the selection criteria of dataset range and model validation). We tested this strategy with water vapor adsorption in a PBR, considering only the transport and sorption kinetic models (13X zeolite), in which adsorption heat causes temperature changes in the PBR. Figure 2b compares the temperature profiles (5 cm from the PBR inlet) predicted by DNMK, which agrees well with the experimental measurements with superior accuracy over the empirical model^[^ ^10^ ^]^ For the ML reaction model, it predicts CO_2_ conversion and H_2_ selectivity for methanol and CO with input parameters of temperature, CO_2_, H_2_, CO, H_2_O and MeOH partial fugacity (5 MPa) ranging from 440–580 K, 6–20, 12–70, 0–5, 0–5 and 0–5, respectively (Figure 2c,d for the validation against experimental measurements on commercial Cu/ZnO/Al_2_O_3_ catalysts^[^ ^39^ ^]^). By integrating the operator‐splitting method with a ML‐trained kinetic model (OS‐ML), this approach accelerates the simulations by ≈750 times compared to a conventional OS model (Figure S2).

### 2.2 Macro‐ and Pores/Particle‐Scale Characterization of SER CO_2_ Hydrogenation

**Figure 3:**
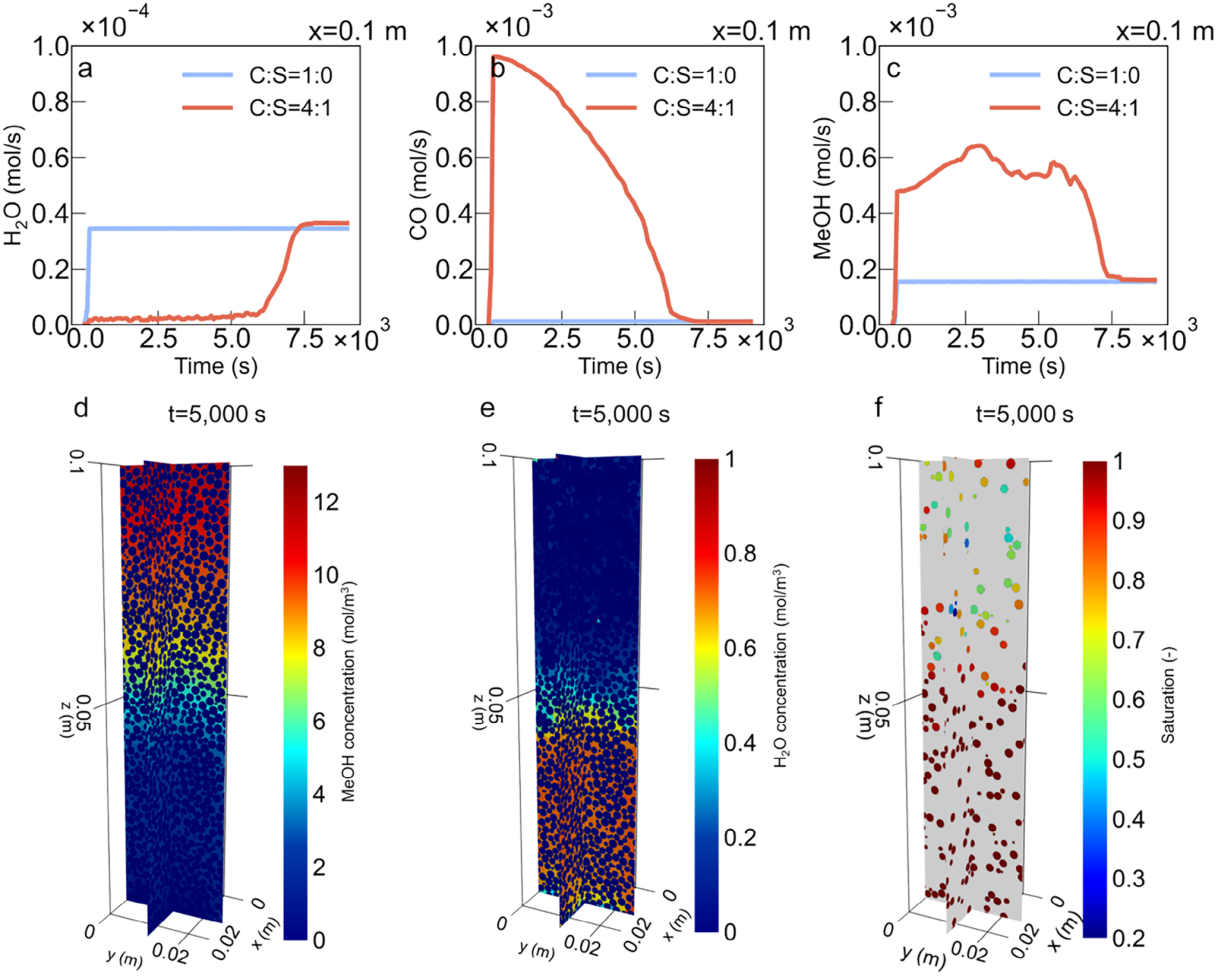
Macro‐ and pores/particle‐scale characterization of SER (C:S = 4:1) and catalyst‐only (C:S = 1:0) systems. Comparison of the product formation rate at the reactor outlet a) H_2_O, b) CO, and c) MeOH. Pore‐scale concentration distribution at t = 5000 s: d) MeOH; and e) H_2_O. f) Pore‐scale saturation of sorbent particles (*ϵ*) at t = 5000 s.

Upon model validation, we applied DNMK to simulate SER CO_2_ hydrogenation in the PBR (random packing) containing 80% catalyst and 20% sorbent particles (C:S = 4:1), and compared it to a catalyst‐only system (C:S = 1:0). **Figure** **3**a–c shows the production rates of CO, methanol (MeOH), and water vapor (H_2_O) at the outlet for both cases (the temporal evolution of species concentrations at different locations are shown in Figure S3, Supplementary Note 3). In the catalyst‐only system (solid blue lines in Figure 3a–c), where water sorption is absent, hydrogenation initiates quickly and reaches steady‐state at t = 180 s. At this point, the MeOH and CO rates stabilize at 1.54 × 10^−4^ and 1.24 × 10^−5^ mol s^−1^, respectively, while H_2_O accumulation levels off at 3.46 × 10^−5^ mol s^−1^. This suppresses MeOH formation as the system approaches equilibrium due to the accumulation of H_2_O (Equations.S9–S12, Supplementary Note 2). Introducing 20% of sorbent particles significantly improves performance of the PBR (solid red lines in Figure 3a–c). The outlet H_2_O concentration drops to almost zero until the sorbent becomes saturated at around *t* = 7500 s (Figure 3a). CO formation rapidly peaks at approximately 0.95 × 10^−3^ mol s^−1^ at around *t* = 400 s (Figure 3b), and MeOH formation is also significantly enhanced (Figure 3c), leading to improved overall CO_2_ conversion (Figure S4). These results align with the kinetic model, where H_2_O removal shifts the thermodynamic equilibrium toward the products, that is, primarily CO (Equations S13 and S14, Supplementary Note 2) and secondarily MeOH, as a significant presence of CO in the system also promotes subsequent CO hydrogenation to MeOH (Equations. S15–S20, Supplementary Note 2) (Figure S5). Overall, the DNMK model accurately captures the macroscopic behavior of SER CO_2_ hydrogenation. The results are consistent with experimental observations reported in the literature using different catalysts and sorbents (e.g., CuInZnZrOx,^[^ ^40^ ^]^ Ru–Na_2_ZrO_3_,^[^ ^41^ ^]^ MOF,^[^ ^42^ ^]^ molecular sieve 3A^[^ ^43^ ^]^) under various conditions.^[^ ^27^, ^33^ ^]^ Note that the total computational time using the ML‐integrated DNMK was approximately 720 h on standard computational resources (AMD Ryzen Threadripper 3970 × 32‐Core Processor), making DNMK a practical solution for practical applications. This highlights that the integration of ML is essential for enabling the practical use of DNMK in industrial contexts.

DNMK also provides pore‐scale insights correlated to PBR performance, which are difficult to interpret directly from experiments. Figure 3d–f shows a snapshot of the MeOH and H_2_O concentration within individual pores and the saturation of individual sorbent particles (ε  = *Q*/Q*, the ratio between the adsorbed amount of water and the equilibrium adsorption capacity, where 1 means fully saturated) at t = 5000 s. The inverse relationship between MeOH and H_2_O concentrations highlights the suppressive effect of H_2_O on MeOH formation, in agreement with the reaction balance (Supplementary Note 2). Specifically, Figure 3d shows that at t = 5000 s, MeOH production in the pore space at the inlet (0–0.05 m) is significantly reduced due to high H_2_O concentration (>0.7 mol m^−3^) (Figure 3e), while regions further away from inlet are still active for MeOH formation until the sorbent particles become saturated (Figure 3f). More importantly, we observe a wide distribution of H_2_O concentration within individual pores (see Figure S6a), indicating different local reaction rates. For each pore, reaction is controlled by the ratio between sorbent‐covered pore surface area and catalyst‐covered pore surface area (*A_s_*/*A_c_*) (Figure S6b), and the saturation of the associated sorbents.

**Figure 4:**
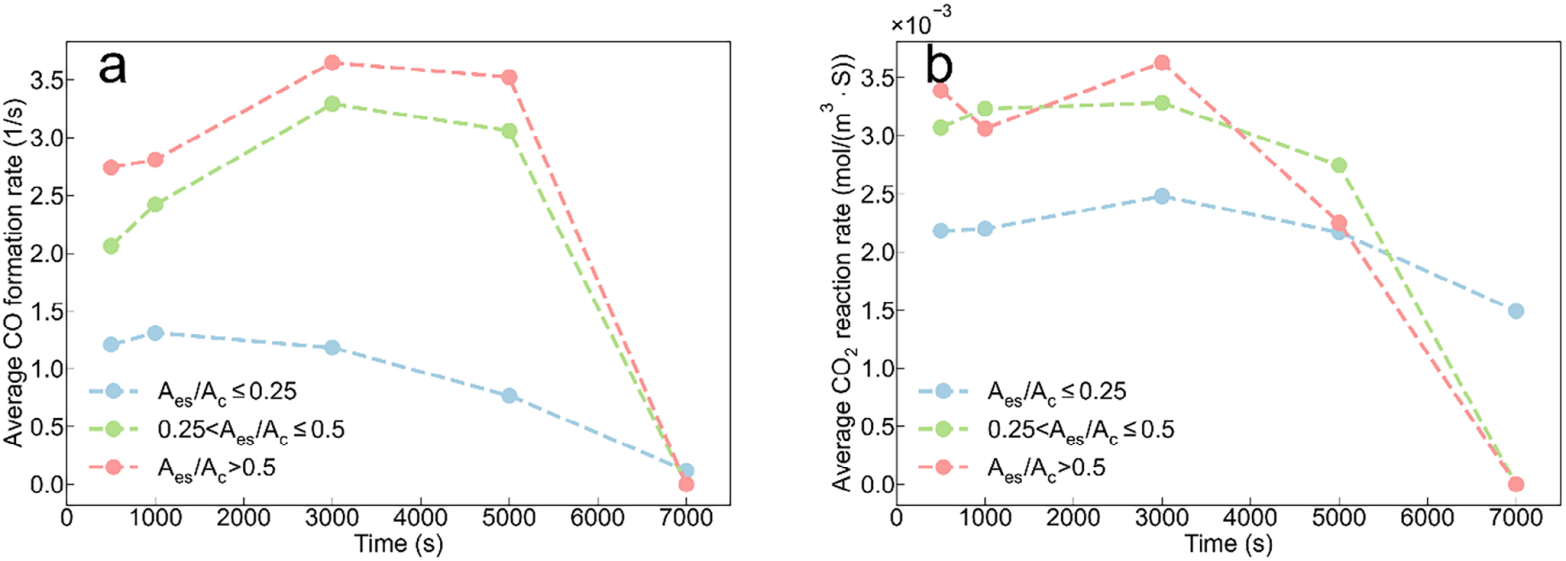
a) Average CO formation rate and b) CO_2_ reaction rate for different *A_es_*/*A_c_* ranges (≤0.25, 0.25–0.5, and >0.5) as a function of time.

To understand how the spatial distribution and saturation of the sorbent particles affect PBR performance, which is essential for the bespoke design of better reactors, we define the effective sorbent to catalyst ratio (*A_es_*/*A_c_*), which is the sorbent to catalyst ratio (*A_s_*/*A_c_*) times sorbent saturation (ε). **Figure** **4**a,b show that *A_es_*/*A_c_* correlates with CO selectivity and CO_2_ conversion: a higher *A_es_*/*A_c_* promotes CO formation and CO_2_ conversion before full sorbent saturation (at ≈6000 s). Specifically, compared to pores with *A_es_*/*A_c_* ≤ 0.25 (indicating 20% sorbent and 80% catalyst), pores with *A_es_*/*A_c_* > 0.25 have significantly higher CO formation and CO_2_ reaction rates, leading to greater MeOH formation. This suggests that a higher proportion of sorbent coverage in a pore helps to maintain low vapor concentrations to create a more ideal local environment for enhancing the apparent catalyst performance.

### 2.3 Exploration of a Design Strategy for SER CO_2_ Hydrogenation PBRs Proposed by DNMK

**Figure 5:**
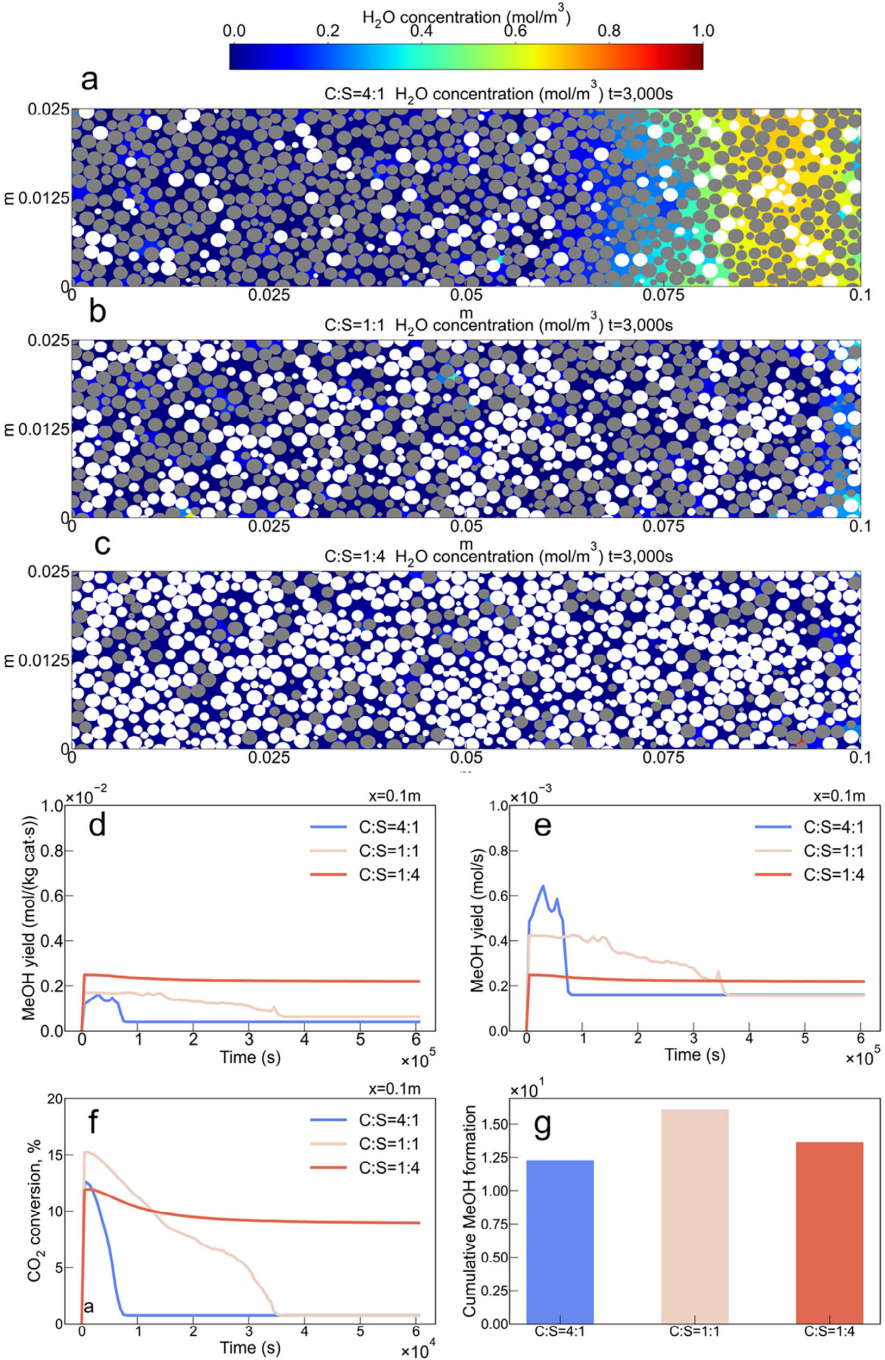
a–c) Snapshots of H_2_O concentration profiles in the reactor pores at different C:S ratios of 4:1, 1:1, and 1:4 (Gray particles represent catalysts, and white particles represent sorbents). d) Comparison of the catalyst mass‐specific product formation rate at the reactor outlet for MeOH. e) Comparison of the absolute MeOH formation rate at the reactor outlet. f) Comparison of CO_2_ conversion at the reactor outlet. g) Comparison of absolute cumulative MeOH formation at the reactor outlet.

We introduce additional sorbent particles into the system to achieve higher sorbent coverage and evaluate the effect of sorbent‐to‐catalyst ratios (C:S) to propose an optimal design strategy using DNMK. The results reveal that increasing the proportion of sorbent particles (C:S = 4:1, 1:1, 1:4) can improve the catalyst performance. This is achieved by reducing local H_2_O concentration in the pore space (see **Figure** **5**a–c at t = 3000 s as an example), delaying H_2_O breakthrough time (Figure S7a), and forming a more suitable micro‐environment for the catalyst. This consequently increases the catalyst mass‐specific product formation for CO (Figure S7b) and MeOH (Figure 5d), in which CO acts as a reactive intermediate (as discussed above). The DNMK reveals that strategic sorbent placement along the reactor length helps to boost catalyst performance and sustain MeOH selectivity, while avoiding early sorbent saturation.

To validate the predicted improvement of catalyst efficiency, we performed laboratory experiments using a CuIn_0.5_/ZnZrO_x_ catalyst and 13X zeolite sorbent (see Figure S8, Supplementary Note 4 for relevant experimental details). We observed increased catalyst mass‐specific CO_2_ conversion (Figure S9a), CO formation (Figure S9b), and MeOH formation (Figure S9c) with increasing the proportion of sorbent particles, aligning with our prediction that more sorbent leads to beter catalytic efficiency.

However, increasing the proportion of sorbent particles introduces a trade‐off between overall reactor performance correlated to the quantity of the catalyst—measured by absolute MeOH production yield—and efficiency of a single catalyst particle, reflected by the catalyst mass‐specific performance metrics discussed above. Although adding more sorbent helps to maintain low vapor concentrations, thereby enhancing local reaction rates at catalyst surfaces, it simultaneously reduces the total available catalyst surface area within the PBR. This, in turn, limits the absolute amount of MeOH that can be produced. As shown in Figure 5e, the C:S = 1:1 case offers a well‐balanced configuration: it substantially extends the duration of the SER effect compared to the C:S = 4:1 case, while achieving a higher MeOH formation rate than the C:S = 1:4 case. This balanced design results in the highest overall CO_2_ conversion rate (Figure 5f) among the three cases and the highest cumulative MeOH production (Figure 5g). These results demonstrate the DNMK's capability of assessing the relevant trade‐offs in catalytic PBRs (due to the presence of various phenomena), which might lead to the formulation of relevant criterions for judging the overall efficiency of the reactor, thus the rational reactor optimization and design strategies.

## 3 Conclusions and Outlook

Accurate interpretation of the coupling between microscopic reaction processes and pore‐scale transport remains one of the foremost challenges in chemical engineering due to the substantial disparity in spatial and temporal scales. Conventional simulation frameworks often fail to capture these multiscale interactions efficiently, limiting their predictive power and their usefulness in guiding rational reactor design. In this work, we introduced DNMK, a data‐driven, multiscale modeling framework that integrates detailed kinetics, pore‐scale transport, and machine‐learning (ML) acceleration to overcome these limitations. The DNMK model offers several key advances of multiscale resolution, enhanced computational efficiency and robust predictive capability, as detailed:

1) DNMK decouples and elucidates the complex interplay between transport and reaction phenomena across scales, enabling more accurate interpretation of experiments and informed design of optimized catalytic systems.
2) By embedding ML surrogate models, DNMK bridges the wide timescale gap between reaction kinetics and reactor‐scale transport, achieving high‐fidelity simulations at a fraction of the computational cost
3) Validated against both experimental measurements and literature benchmarks, DNMK reliably reproduces reactor‐scale dynamics and pore‐level behavior, making it broadly applicable for both fundamental studies and industrial process modeling.

Although this study focuses on the SER CO_2_ hydrogenation process (without phase change) using a Cu/ZnO/Al_2_O_3_ catalyst and a 13X sized for small to moderate reactor scales (up to the centimeter scale), the underlying framework is readily generalizable. The demonstrated ability of DNMK to narrow design spaces and identify optimal operational regimes already represents a significant step toward predictive, data‐driven reactor engineering for practical applications. Future work will extend DNMK to include phase‐change processes, more advanced ML surrogates, and complex flow regimes at higher Reynolds numbers, as well as to accelerate computation for larger‐scale systems. With these advances, DNMK has the potential to evolve into a scalable, adaptive, and computationally efficient platform for the bottom‐up design and optimization of catalytic reactors, ultimately enabling more sustainable and efficient chemical manufacturing.

## 4 Experimental Section

### Construction of the Dual‐Network Model (DNM)

The open‐source software LIGGGHTS (www.liggghts.com) employing the Discrete Element Method (DEM) generated a packed bed consisting of 36390 randomly packed spheres (with a uniform diameter of 2 mm), which occupies a volume of 0.025 × 0.025 × 0.1 m^3^ with a bed porosity of 0.36, as shown in Figure 2a. Details of the method for generating the DNM model are described in our previous study.^[^ ^44^ ^]^ In brief, the construction of the DNM involves applying a network extraction method to images of porous media to generate a topologically equivalent network, where wide regions are represented as nodes connected by throats or links, capturing the pore space, solid phase, and their interfaces. From this network, structural information, including the volumes of pore and solid nodes, interfacial contact areas, and connectivity, is extracted directly from the real geometry, ensuring an accurate representation of the connectivity, tortuosity, and heterogeneity of the pore space. The DNM allows calculation of node surface areas, equivalent radii, and volumes, identification of neighboring nodes and associated links, as well as determination of link lengths and cross‐sectional (interfacial) areas between nodes, preserving the geometric and topological features essential for simulating fluid flow, transport, and reaction processes. The length of the links was defined as the distance between the centers of two adjacent nodes, which can be the pore‐pore, pore‐solid, or solid‐solid connections.

### Flow Model

In the DNM, fluid flow and heat transfer were described by conservation equations for mass and energy. Considering node *i* with *N_j_* connecting nodes, mass balance gives

(1) $$\sum_{j=1}^{N_{j}}q_{ij}\rho_{f}=\Delta V^{j}\rho_{f}+\delta V^{j}\rho_{f}$$

where *q_ij_* is the volumetric flowrate (m^3^/s) in the link of *ij* which connects node *i* and node *j*, Δ*V^j^* is the change of volume relying on molar change, δ*V^j^* is the difference of volume between actual volume of the pore and the volume of the gas phase while *N_j_* is the number of links; ρ_*f*_ is the fluid density (kg/m^3^).

The pressure drop for laminar flow between node *i* and *j* is.^[^ ^45^ ^]^

(2) $$P_{i}-\;P_{j}=\left(\frac{1}{g_{ij}}+E_{ij}+C_{ij}\right)q_{ij}\;$$

where *P_i_* and *P_j_* are the pressure (Pa) of void node *i* and *j*, respectively; 1/*g_ij_* is the resistance coefficient of fluid flow in the link, defined as:

(3) $$\frac{1}{g_{ij}}=\frac{\mu l_{ij}}{k{A_{ij}}^{2}G}\;$$

where the constant *k* is 0.5, 0.6 and 0.5623 for a circular, an equilateral and a square cylinder, respectively, depending on the value of *G*, which is the shape factor defined according to the literature,^[^ ^46^ ^]^ *A_ij_* is the cross‐sectional area of the link, and µ is the viscosity of the fluid (Pa·s) in **Table** **1**.

**Table 1:** Input parameters for the DNMK.

Parameter	Value	Parameter	Value
*L*	0.10 m	*W*	0.025 m
*H*	0.025 m	*c*_*p*,*f*_	1013 J/(kg·K)
λ_*s*_	0.4 W/(K·m)	*c*_*p*,*sor*_	880 J/(kg·K)
λ_*f*_	0.03 W/(K·m)	*c*_*p*,*cat*_	1045 J/(kg·K)
*T_i_*	291 K	ρ_*air*_	1.205 kg m^−3^
*d*	0.002 m	ρ_*sor*_	1,150 kg m^−3^
*M*_*N*2_	0.028 kg mol^−1^	ρ_*cat*_	1,450 kg m^−3^
*M*_*H*2*O*_	0.018 kg mol^−1^	Δ*E*	65572 J mol^−1^
*M_CO_*	0.028 kg mol^−1^	k*	7
*M*_*CO*2_	0.044 kg mol^−1^	µ	1.8205 × 10^−5^ Pa·s
*M*_*H*2_	0.002 kg mol^−1^	*b*_0_	4.002 Pa^−1^
*M_Me_*	0.032 kg mol^−1^	*n*_0_	2.976
*q_max_*	17 mol kg^−1^	α	0.377

The expansion frictional loss factor *E_ij_* in (Equation 2) can be expressed by Equation (4).^[^ ^45^ ^]^

(4) $$E_{ij}=\left[\frac{\rho_{f}q_{ij}}{2\pi^{2}{A_{ij}}^{2}}\left[{\left(\frac{E_{0}}{Re_{ij}}\right)}^{m}+{\left(1-{\left(\frac{r_{ij}}{r_{j}}\right)}^{2}\right)}^{2m}\right]-\frac{\gamma }{2\pi^{2}}\rho_{f}q_{ij}\left(\frac{1}{r_{ij}^{4}}-\frac{1}{r_{j}^{4}}\right)\right]\;$$

where *E*_0_ is the laminar constant for expansion which is 27, and *m* is curvature factor of expansion which is 1.1; *Re_ij_* is Reynolds number of fluid in link *ij*; *r_ij_* is the effective radius (m) of link *ij*; *r_j_* is the effective radius of node *j*; γ is a flow pattern constant, which is set to 1 as a flat velocity profile is assumed.

Moreover, *C_ij_* is the contraction fractional loss factor in (Equation 2)^[^ ^45^ ^]^:

(5) $$\begin{array}{c} C_{ij}=\left[\;\frac{\rho_{f}q_{ij}}{2\pi^{2}{A_{ij}}^{2}}\left[\;{\left(\;\frac{C_{0}}{Re_{ij}}\;\right)}^{n}+{\left(\;\left(1-{\left(\;\frac{r_{ij}}{r_{i}}\;\right)}^{2}\;\right)/2\;\right)}^{n}\;\right]-\frac{\gamma }{2\pi^{2}}\rho_{f}q_{ij}\left(\frac{1}{r_{i}^{4}}-\frac{1}{r_{ij}^{4}}\right)\right] \end{array}$$

where *C*_0_ is the laminar constant for contraction that is 26 in a wide range of *Re_ij_* between 10 and 1 × 10^4^, and *n* is 0.8 which is the curvature parameter of contraction. *Re_ij_* in (Equations 4 and 5) is defined by

(6) $$Re_{ij}=\frac{2\rho_{f}q_{ij}}{\pi \mu r_{ij}}\;$$

In this work, the mixture of air and water vapor is assumed to be an ideal gas without phase change

(7) P=cRT

where *P* is the pressure (Pa), *c* is the molar concentration (mol/m^3^) and *R* is the ideal gas constant (J/(mol·K)).

The density of fluid in the node *i* is calculated by:

(8) $$\rho_{f,i}=\frac{P_{i}\sum_{i}^{N}c_{i}M_{i}}{RT\sum_{i}^{N}c_{i}}\;$$

where *c_i_* and *M_i_* is the molar concentration (mol/m^3^) and molar mass (kg/mol) of component *i*, respectively.

The pressure within each pore is assumed to be in equilibrium to enhance numerical stability. In the pore space, gas can be generated or consumed due to reaction. Hence, according to the ideal gas assumption, gas expansion or contraction may occur in the pore space, and the excess or insufficient part can then leave or replenish through flow. The corresponding volume change is:

(9) $$\left\{\begin{array}{cc} & \Delta \;V^{j}=\sum_{i}^{N}\Delta n_{i}\;R\frac{T^{j}}{P^{j}}\\ & \delta \;V^{j}=R\frac{T^{j}}{P^{j}}\sum_{i}^{N}n_{i}-V_{a}^{j} \end{array}\right.$$

where *n_i_* is the moles of gas component *i*, *N* is the number of gas components in the pore space, and $V_{a}^{j}$ is the volume of pore *j*.

### Advection Diffusion and Convection Heat Transfer

Transport of gas components within a PBR is governed by advection and diffusion:

(10) $$\frac{\partial \left(c_{w}\right)}{\partial t}+\nabla \left(vc_{w}\right)-\nabla \cdot \left(D_{w}\nabla c_{w}\right)=\rho_{s}\;\frac{\partial Q}{\partial t}+S_{w}$$

where *c_w_* is the concentration of water (mol/m^3^), *v* is the velocity in the pores (m/s), *D_w_* is the moisture diffusion coefficient (m^2^/s), *Q* means the adsorbent loading (mol/kg), ρ_*s*_ is density of solid (kg/m^3^) and *S_w_* is the source term of H_2_O conversion based on reaction that is defined in Supplementary Note 2 (Equation S28). Except for H_2_O, the mass balance for component *i* is:

(11) $$\frac{\partial \left(c_{i}\right)}{\partial t}+\nabla \left(vc_{i}\right)-\nabla \cdot \left(D_{i}\nabla c_{i}\right)=S_{i}\;$$

where *c_i_* is the concentration of component *i* (mol/m^3^) and *D_i_* is the pore diffusivity^[^ ^9^ ^]^ of component *i* (m^2^/s) and *S_i_* is the source term of species conversion (Supplementary Note 2).

For heat transfer within a PBR, we applied energy conservation considering both heat convection and conduction. For the heat transfer in the fluid phase, convection and conduction are considered:

(12) $$\frac{\partial \left(\rho_{f}c_{p,f}T_{f}\right)}{\partial t}+\nabla \left(\rho_{f}c_{p,f}T_{f}v\right)-\nabla \cdot \left(\lambda_{f}\nabla T_{f}\right)-h_{sf}\;\left(T_{s}-T_{f}\right)=0$$

where ρ_*f*_ is the fluid density (kg/m^3^), *c*_*p*,*f*_ is the specific heat capacity of fluid (J/(kg·K)), *T_s_* and *T_f_* are the temperature of solid and fluid (K), respectively, λ_*f*_ is the thermal conductivity of fluid (W/(m·K)) and *h_sf_* is the convection heat transfer coefficient (W/K). In this work, the heat of reaction is ignored.^[^ ^33^ ^]^

The energy conservation equation for the solid phase can be expressed by:

(13) $$\frac{\partial \left(\rho_{s}c_{p,s}T_{s}\right)}{\partial t}-\nabla \cdot \left(\lambda_{s}\nabla T_{s}\right)-h_{sf}\;\left(T_{f}-T_{s}\right)=\rho_{s}\;\frac{\partial Q}{\partial t}\Delta H$$

where *c*_*p*,*s*_ is the specific heat capacity of solid (J/(kg·K)), λ_*s*_ is thermal conductivity (W/(m·K)) and Δ*H* is the isosteric heat of adsorption (kJ/mol, to be defined later, Equation 19).

### The Kinetic Model for Water Vapor Adsorption and Machine Learning

The adsorbent capacity *Q*(*x*, *t*) is described by the Linear Driving Force (LDF) model^[^ ^30^, ^31^ ^]^ with associated parameters presented in Table 1.

(14) $$\frac{\partial Q}{\partial t}=k_{LDF}^{*}\;\left(Q^{*}-Q\right)$$

where Q* is the equilibrium adsorption capacity (mol/kg), and $k_{LDF}^{*}$ is the adsorption rate constant.

(15) $$k_{LDF}^{*}=\frac{k^{*}}{\rho_{s}RT_{s}\frac{\partial Q^{*}}{\partial P_{w}}}\;$$

where k* is an empirical constant set as 7, and Q* was obtained from the Langmuir‐Freundlich model:

(16) $$Q^{*}=\frac{Q_{max}{\left(bP_{w}\right)}^{1/n}}{1+{\left(bP_{w}\right)}^{1/n}}\;$$

where *Q_max_* is the maximum amount of the adsorbed water vapor in the sorbent (mol/kg), and *P_w_* is partial pressure of the water vapor which is defined by the state equation (Equation 11), while *b* and *n* are parameters of the model, defined as:

(17) $$b=b_{0}\;exp\left(\frac{\Delta E}{RT_{0}}\left(\frac{T_{0}}{T}-1\right)\right)$$

(18) $$\frac{1}{n}=\frac{1}{n_{0}}+\alpha \left(1-\frac{T_{0}}{T}\right)$$

where *n*_0_, *b*_0_, Δ*E*, *T*_0_ and α are empirical constants. The isosteric heat of the adsorption Δ*H* (J/mol) in (Equation 13) is then calculated using

(19) $$\Delta H=\Delta E-\alpha RT_{0}n^{2}\ln\left(\frac{Q}{Q_{max}-Q}\right)$$

The timescale of the sorption process (≈10^−5^ s) is much lower than that of heat/mass transfer (≈10^−1^ s), accordingly, computation of the governing equations (Equations 10 and 13) together with the adsorption source term is expensive. Operator splitting (OS) was employed to address this issue. As shown in Figure 2, at each time step, the partial differential equations (Equations 10 and 13) were solved initially without the source term (i.e., the right‐hand sides). Then, after updating the temperature/concentration profiles, the following ordinary differential equations were solved by the fourth‐order Runge–Kutta method using the SCIPY package in PYTHON:

(20) $$\left\{\begin{array}{cc} & \frac{\partial Q}{\partial t}=k_{LDF}^{*}\;\left(Q^{*}-Q\right)\\ & V_{v}\;\frac{\partial c_{w}}{\partial t}=V_{s}\;\rho_{s}\frac{\partial Q}{\partial t}\\ & \rho_{s}c_{p,s}\;\frac{\partial T_{s}}{\partial t}=\rho_{s}\;\frac{\partial Q}{\partial t}\Delta H \end{array}\right.$$

To improve the efficiency of simulating the sorption process, machine learning (ML) can be employed. Solving (Equations 20) for each contact area between the particle surface and the void space is computationally demanding. Consequently, ML was used to speed up this process. In detail, we solved (Equation 20) under various conditions (temperature range: 283.15–530.15 K, concentration range: 0.001–1.6 mol m^−^^3^, adsorption capacity range: 0–17.5 mol kg^−1^) to produce data (such as temperature, concentration of water, and adsorption capacity) to train an ExtraTrees ML model^[^ ^35^ ^]^ before solving those ODEs. As shown in Figure S2, the ML model reproduces the results of the ODE solver, enabling much faster computation.

### The Kinetic Model for CO_2_ Hydrogenation

Methanol synthesis via CO_2_ hydrogenation can be described by the kinetic model developed by Graaf et al.,^[^ ^32^ ^]^ please refer to Supplementary Note 2.

### Numerical Solutions

Strang splitting was employed to decouple the reaction and transport modules operating at different time scales, achieving second‐order accuracy with minimal splitting errors^[^ ^47^ ^]^

(21) $$Va^{n+1}=L_{a}\;\left(\frac{\Delta t}{2}\right)L_{k}\left(\Delta t\right)L_{a}\left(\frac{\Delta t}{2}\right)Va^{n}$$

where *Va^n^* represents the thermodynamic variables at the *n^th^* time step, *L_a_* is the advection‐dispersion operator (calculating temperature and concentration), *L_k_* is the operator of the kinetic model, and Δ*t* is the time step. The time‐step duration (≈0.1 s in this study) was selected to balance computational efficiency and accuracy. The stability and accuracy of the operator‐splitting scheme were verified by comparing simulation results with experimental data, ensuring that the adopted time‐step size introduced no significant deviation in temperature or conversion predictions.

The overall workflow is described below:

- Step 1: We set the initial temperature (*T_i_*) for all nodes and the fluid density in the pores.
- Step 2: We then obtain the predictor to replace the sorption model from the machine learning method, using a time step (Δ*t*/2). This splitting technique is used because adsorption, convection, and diffusion occur at significantly different rates. After that, the reaction model was solved by an ODE solver using a time step (Δ*t*/2) directly. The results from two kinetic models are used to update the temperature, density, and component concentration fields.
- Step 3: Use (Equations (2), (3), (4), (5), (6), (7), (8), (9), (10)) to determine the distribution of density and pressure. To measure the convergence of successive iterations, we calculate the absolute average deviation:
(22) $$AAD=\frac{\sum_{i=1}^{N}\left|P_{inital}\left(i\right)-P_{new}\left(i\right)\right|}{N}\;$$

where *N* is the total number of nodes (both void and solid nodes) in the domain, *i* is the node label, *P_inital_* is the result of the last iteration, and *P_new_* is the current pressure field. The simulation was continued until the AAD was less than 1 × 10^−3^ Pa. Subsequently, (Equations (11), (12), (13)) were solved without the source term to obtain the temperature and concentration distribution for a time step Δ*t*.

- Step 4: Based on the results obtained in Step 3, we repeated Step 2 to refine the density, concentration and temperature distribution.

### Boundary Conditions

The boundary conditions for fluid flow simulation were: atmospheric pressure at the outlet, and 5 × 10^6^ Pa (50 bar) in the PBR. The initial state of the dry feed at the inlet was 505.15 K and 0.077 m s^−1^, and an inlet component concentration of CO_2_ = 226 mol m^−3^, H_2_ = 842 mol m^−3^, and N_2_ = 129 mol m^−3^. The initial temperature of the solid phase was 505.15 K. No‐slip flow conditions were applied to the walls of the PBR and the surface of the solid phase. The initial temperature of the fluid in the PBR was 505.15 K. Initially, the fraction of adsorbed water vapor throughout the solid phase was zero. The initial temperature of the solid was 505.15 K. During the simulation, water was produced via the chemical reactions (Supplementary Note 2), which were adsorbed by the sorbent particles. The simulation stops when full saturation of the sorbent particles in the PBR is achieved.

### Methods—Pre‐Processing

To evaluate the prediction accuracy of CO_2_/H_2_ conversion and MeOH/CO selectivity between the ML‐predicted and literature‐reported values, the accuracy for each data point was calculated as one minus the absolute discrepancy between the predicted and reported value, normalized by the reported value. The average accuracy and corresponding standard deviation were then obtained across all data points.

### Methods—Data Presentation

All quantitative results are reported as mean ± standard deviation (SD). Exact sample sizes (n) are specified in the main text and/or figure captions.

### Methods—Software

Data processing was carried out in Microsoft Excel (2024). All figures were generated using PYTHON's Matplotlib (3.7.3), Plotly (5.22) and Seaborn (0.12.2) packages.

## Conflict of Interest

The authors declare no conflict of interest.

## Supporting information



Supporting Information

## Data Availability

The data and associated code used in the manuscript are available on Github: https://github.com/iPMLab/Multi-physics-Network-Model.
