# Supplementary material for: A Machine Learning‐Driven Pore‐Scale Network Model Coupling Reaction Kinetics and Interparticle Transport for Catalytic Process Design
Source: Adv Sci (Weinh). 2025 Dec 3;13(8):e13649. doi: 10.1002/advs.202513649 (PMC12884819; doi:10.1002/advs.202513649)
Supplement: Supplementary file 1 — Supporting Information [file ADVS-13-e13649-s001.docx]

**A Machine Learning-Driven Pore-Scale Network Model Coupling Reaction Kinetics and Interparticle Transport for Catalytic Process Design**

**SUPPLEMENTARY INFORMATION**

**Supplementary Note 1**

**Machine learning (ML) powered multi-scale modelling**

The sorption model is a high-order nonlinear ODE, which requires substantial computational time when solved using conventional Runge-Kutta methods. To address this, we employed ML. We generated a dataset of 100,000 precomputed samples (temperature range: 283.15–530.15 K, concentration range: 0.001–1.6 mol/m^3^, adsorption capacity range: 0–17.5 mol/kg, vapor partial pressure range: 2.35–7052.27 Pa)^1–3^, with 80,000 samples used for training and 20,000 for testing. The samples cover the reaction conditions that are within the applicable range of the microkinetic model, and include the general operation conditions of the CO_2_ hydrogenation for methanol synthesis, that is, 5 MPa total pressure and 483–553 K temperature^1–3^_._ For each data point, temperature, concentration, and adsorption capacity were randomly selected within their respective ranges to generate the corresponding precomputed results, ensuring that all relevant operating conditions were represented. The ExtraTrees random forest machine learning model was utilized for prediction^4^. The full dataset of 100,000 precomputed samples was generated in approximately 2 hours.

We employed a 5-fold cross-validation strategy to assess the predictive performance of the ML model. The complete dataset, consisting of 100,000 samples of ΔQ, ΔT, and ΔC_w_, was randomly partitioned into five folds (20,000 samples each). In each iteration, one fold was used as the test dataset, while the remaining four (80,000 samples) served as the training data. The ML model trained to predict ΔQ, ΔT, and ΔC_w_ over Δt = 0.05 s, and the predicted values were compared against those computed from the kinetic model. This process was repeated five times, ensuring that each subset is used once for testing. The coefficient of determination (R^2^) values across all 100,000 samples are 0.961 for ΔQ, 0.942 for ΔT, and 0.989 for ΔC_w_ (**Fig. S1a–S1c**), demonstrating the high predictive accuracy and generalization capability of the ML model. In addition, as shown in **Fig. S1d–S1e**, the MSE for the training learning curve and test learning curve stay below 0.02 with 80,000 training datasets, indicating sufficient accuracy for the ML surrogate model.

**
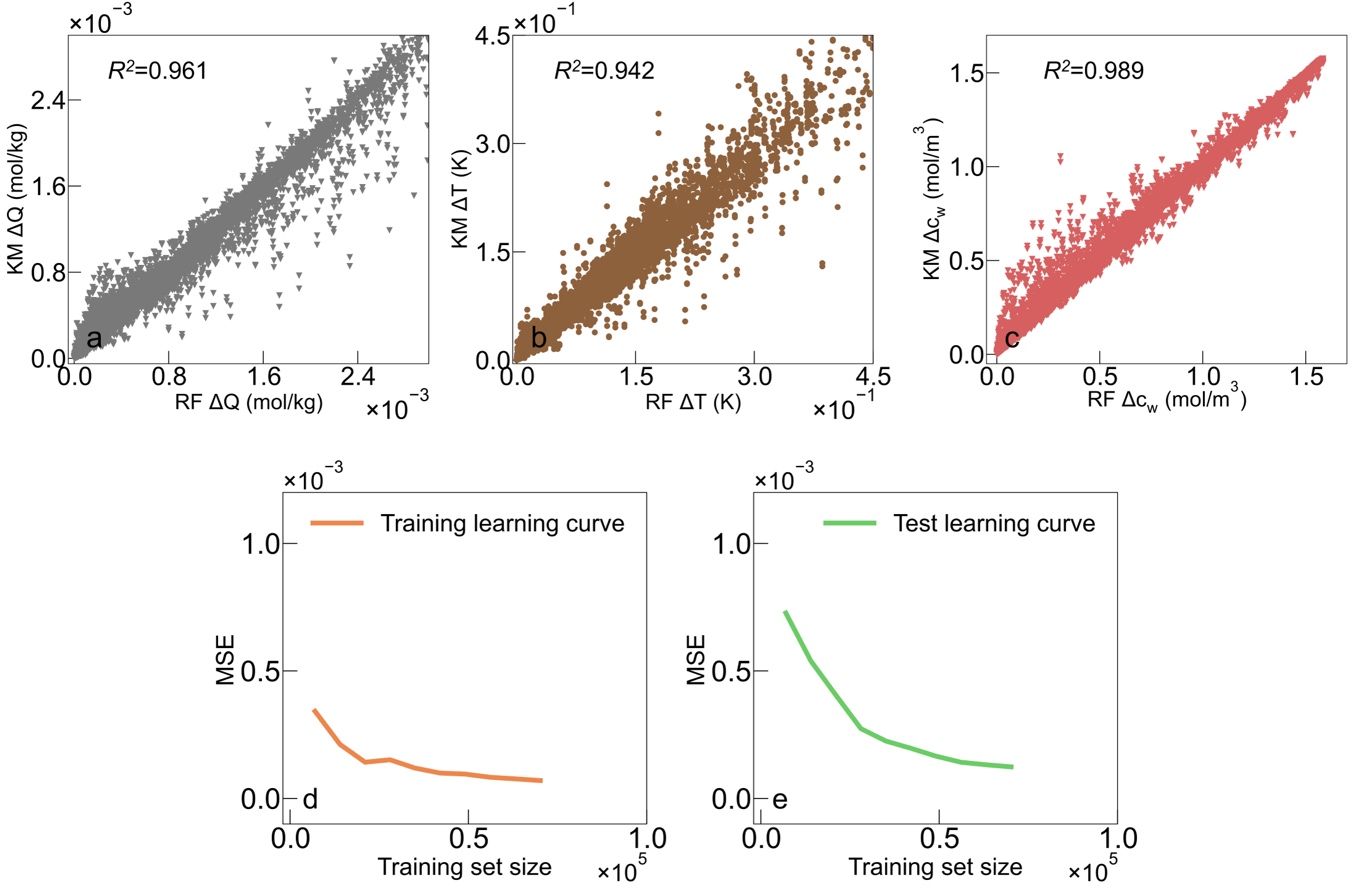
**

**Fig. S1.** Comparison of kinetic model (KM) results and random forest (RF) predictions for: (a-c) change of adsorption capacity (*ΔQ*), temperature (*ΔT*) and water vapor concentration (*ΔC_w_*) over *Δt* = 0.05 s; (d) Mean squared error (MSE) for the training learning curve; (e) Mean squared error (MSE) for the test learning curve.

**Fig. S2a** compares the computational time required for a single step, with and without the application of machine learning. **Fig. S2b** illustrates the temporal resolution achieved using OS-ML compared to the conventional approach.

**
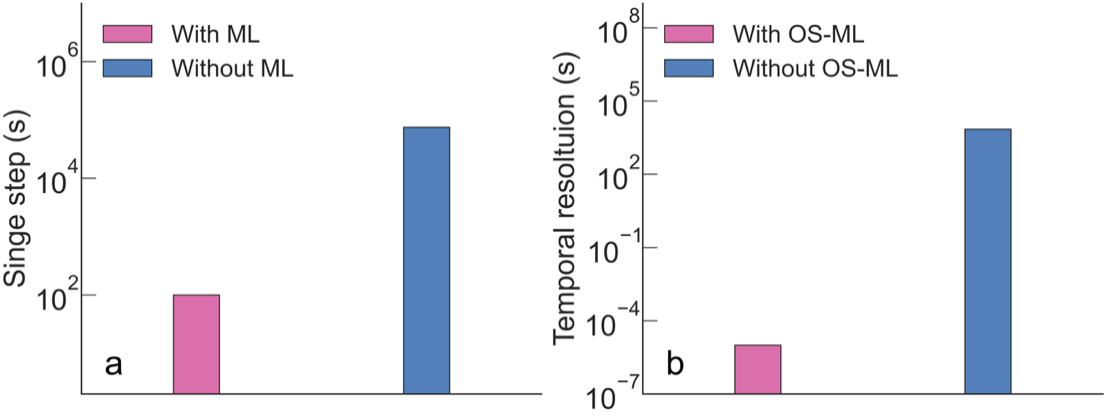
**

**Fig. S2.** The comparison of performance for (a) with machine learning (ML) and without ML and (b) with operator splitting machine learning (OS-ML) and without OS-ML.

**Supplementary Note 2**

**Kinetic models for water vapor sorption**

In this work, there are comprehensive validations of our model from both physical and chemical perspectives. For physical processes including transport and sorption, the laboratory-scale sorption experiment (in a cylindrical packing of length 10 cm, and diameter 3.8 cm) and an analytic solution from Gao et al.^5^ were selected as validation. Here, the DNMK was used to simulate physical processes (transport and sorption) within individual pores in this system for validation (under the initial condition of inlet flow velocity = 0.21 m/s, the inlet water vapor partial pressure = 1,325 Pa, and the initial temperature = 293.15K).

**The kinetic model for CO_2_ hydrogenation.**

For the chemical process of CO_2_ hydrogenation, a tubular reactor experiment from Portha et al.^3^ was selected as validation. Here the reactor has an external diameter of 1.27 cm, while the internal diameter was 1.01 cm. Powdered commercial Cu/ZnO/Al_2_O_3_ catalyst (mass: 135 mg) was used with a gas hourly space velocity (volumetric flow rate to the total volume of the PRB) of 7800 h^−1^, with varying H_2_/CO_2_ ratios from 2 to 6. The conversion rates of H_2_ and CO_2_, as well as the selectivity for CO and methanol works as benchmark. The CO_2_ and H_2_ conversions, as well as MeOH and CO selectivity are:

|  | $X_{\mathrm{CO}_{2}}=\frac{n_{CH_{3}\mathrm{OH}}+n_{\mathrm{CO}}}{n_{{CO}_{2}, initial}}$ | (S1) |
| --- | --- | --- |
|  | $X_{H_{2}}=\frac{{2n}_{CH_{3}\mathrm{OH}}+n_{H_{2}O}}{n_{H_{2}, initial}}$ | (S2) |
|  | $S_{CH_{3}\mathrm{OH}}=\frac{n_{CH_{3}\mathrm{OH}}}{n_{CH_{3}\mathrm{OH}}+n_{\mathrm{CO}}}$ | (S3) |
|  | $S_{\mathrm{CO}}=\frac{n_{\mathrm{CO}}}{n_{CH_{3}\mathrm{OH}}+n_{\mathrm{CO}}}$ | (S4) |

The full reaction network from Graaf et al.^1^ is shown below, where s1 and s2 mean reaction sites.

| **Adsorption equilibria** | | | | | |
| --- | --- | --- | --- | --- | --- |
|  | $CO+s1=CO\_s1$ | | | (S5) | |
|  | $CO_{2}+s1=CO_{2}\_s1$ | | | (S6) | |
|  | $H_{2}+2s2=2H\_s2$ | | | (S7) | |
|  | $H_{2}O+s2=H_{2}O\_s2$ | | | (S8) | |
| **Reaction A** | | | | | |
| A1 | | $CO\_s1+H\_s2=HCO\_s1+s2$ | (S9) | |  |
| A2 | | $HCO\_s1+H\_s2=H_{2}CO\_s1+s2$ | (S10) | |  |
| A3 | | $H_{2}CO\_s1+H\_s2=H_{3}CO\_s1+s2$ | (S11) | |  |
| A4 | | $H_{3}CO\_s1+H\_s2=CH_{3}OH+s1+s2$ | (S12) | |  |
| **Reaction B** | | | | |  |
| B1 | $CO_{2}\_s1+H\_s2=HCO_{2}\_s1+s2$ | | | (S13) | |
| B2 | $\mathrm{HC}O_{2}\_s1+H\_s2=CO\_s1+H_{2}O\_s2$ | | | (S14) | |
| **Reaction C** | | | | | |
| C1 | $CO_{2}\_s1+H\_s2=HCO_{2}\_s1+s2$ | | | (S15) | |
| C2 | $\mathrm{HC}O_{2}\_s1+H\_s2=H_{2}CO_{2}\_s1+s2$ | | | (S16) | |
| C3 | $H_{2}CO_{2}\_s1+H\_s2=H_{3}CO_{2}\_s1+s2$ | | | (S17) | |
| C4 | $H_{3}CO_{2}\_s1+H\_s2=H_{2}CO\_s1+H_{2}O\_s2$ | | | (S18) | |
| C5 | $H_{2}CO\_s1+H\_s2=H_{3}CO\_s1+s2$ | | | (S19) | |
| C6 | $H_{3}CO\_s1+H\_s2=\mathrm{CH}_{3}OH+s1+s2$ | | | (S20) | |

To summarize, we have:

|  | $CO+2H_{2}\rightleftharpoons\mathrm{CH}_{3}\mathrm{OH}$ | (S21) |
| --- | --- | --- |
|  | $\mathrm{CO}_{2}+H_{2}\rightleftharpoons CO+H_{2}O$ | (S22) |
|  | $\mathrm{CO}_{2}+3H_{2}\rightleftharpoons\mathrm{CH}_{3}OH+H_{2}O$ | (S23) |
|  | $R_{MeOH, A2}=\frac{k_{1}K_{\mathrm{CO}}\left( f_{\mathrm{CO}}f_{H_{2}}^{1.5}-\frac{f_{CH_{3}\mathrm{OH}}}{\sqrt{f_{H_{2}}}K_{1}^{0}} \right)}{\left( 1+K_{\mathrm{CO}}f_{\mathrm{CO}}+K_{CO_{2}}f_{CO_{2}} \right)\left[ \sqrt{f_{H_{2}}}+\frac{K_{H_{2}O}f_{H_{2}O}}{\sqrt{k_{H_{2}}}} \right]}$ | (S24) |
|  | $R_{H_{2}O,B2}=\frac{k_{2}K_{CO_{2}}\left( f_{CO_{2}}f_{H_{2}}-\frac{f_{H_{2}O}f_{\mathrm{CO}}}{K_{2}^{0}} \right)}{\left( 1+K_{\mathrm{CO}}f_{\mathrm{CO}}+K_{CO_{2}}f_{CO_{2}} \right)\left[ \sqrt{f_{H_{2}}}+\frac{K_{H_{2}O}f_{H_{2}O}}{\sqrt{k_{H_{2}}}} \right]}$ | (S25) |
|  | $R_{MeOH, C2}=\frac{k_{3}K_{CO_{2}}\left( f_{CO_{2}}f_{H_{2}}^{1.5}-\frac{f_{CH_{3}\mathrm{OH}}f_{H_{2}O}}{\sqrt{f_{H_{2}}}K_{3}^{0}} \right)}{\left( 1+K_{\mathrm{CO}}f_{\mathrm{CO}}+K_{CO_{2}}f_{CO_{2}} \right)\left[ \sqrt{f_{H_{2}}}+\frac{K_{H_{2}O}f_{H_{2}O}}{\sqrt{k_{H_{2}}}} \right]}$ | (S26) |

where *R*_j_ is the rate (mol/(kg·s)) of reaction *j*; *k* is the kinetic constant for reaction for catalyst mass (mol/(s·Pa·kg_cata_)); *K* is the component adsorption equilibrium constant (Pa^-1^), and *f* is the component partial fugacity (Pa). The constants of reaction are given by the Arrhenius equation:

|  | $\left\{ \begin{aligned} k_{i}=A_{i}exp\left( \frac{-E_{a,i}}{RT} \right) \\ K_{j}=A_{j}exp\left( \frac{-\Delta H_{ads,j}^{cat}}{RT} \right) \end{aligned} \right.$ | (S27) |
| --- | --- | --- |

where *A* is pre-exponential factor; $E_{a}$ is activation energy of reaction (J/mol); $\Delta H_{ads}^{cat}$ is component heat of adsorption (J/mol). The changes of components are given as:

|  | $S_{i}V_{v}=V_{v}\frac{\partial c_{i}}{\partial t}=m_{cata}\sum\vartheta_{i}R_{i}$ | (S28) |
| --- | --- | --- |

where $\vartheta$ is the stoichiometric coefficient, $m_{cata}$ is the mass of catalyst (kg). The kinetic parameters for CO_2_ hydrogenation in Eqs. S24–27 are given in **Table S1**.

**Table S1.** Kinetic parameter values for CO_2_ hydrogenation by Maksimov et al.^6^

| Parameter | Value |
| --- | --- |
| $A_{1}$, mol/(s kg_cat_ Pa) | 0.8303×10^−5^ |
| $A_{2}$, mol/(s kg_cat_ Pa) | 3.24×10^−3^ |
| $A_{3}$, mol/(s kg_cat_ Pa) | 0.1957×10^−5^ |
| $A_{\mathrm{CO}}$, Pa^−1^ | 5.10×10^−9^ |
| $A_{CO_{2}}$, Pa^−1^ | 4.90×10^−7^ |
| $A_{H_{2}/H_{2}O}$, Pa^−1^ | 3.18×10^−15^ |
| $E_{a,1}$, J/mol | 3.22×10^4^ |
| $E_{a,2}$, J/mol | 6.47 × 10^4^ |
| $E_{a,3}$, J/mol | 2.52×10^4^ |
| ${\Delta H}_{ads,\mathrm{CO}}^{cat}$, J/mol | −1.34×10^4^ |
| ${\Delta H}_{ads,\mathrm{CO}_{2}}^{cat}$, J/mol | −1.26×10^3^ |
| ${\Delta H}_{ads,{H_{2}}/{H_{2}O}}^{cat}$, J/mol | −1.01×10^5^ |

**Supplementary Note 3**

The comparison of temporal evolution of the rate of species of the SER (C:S = 4:1) and catalyst-only (C:S = 1:0) systems at different locations of the PBR are shown in **Fig. S3**

**
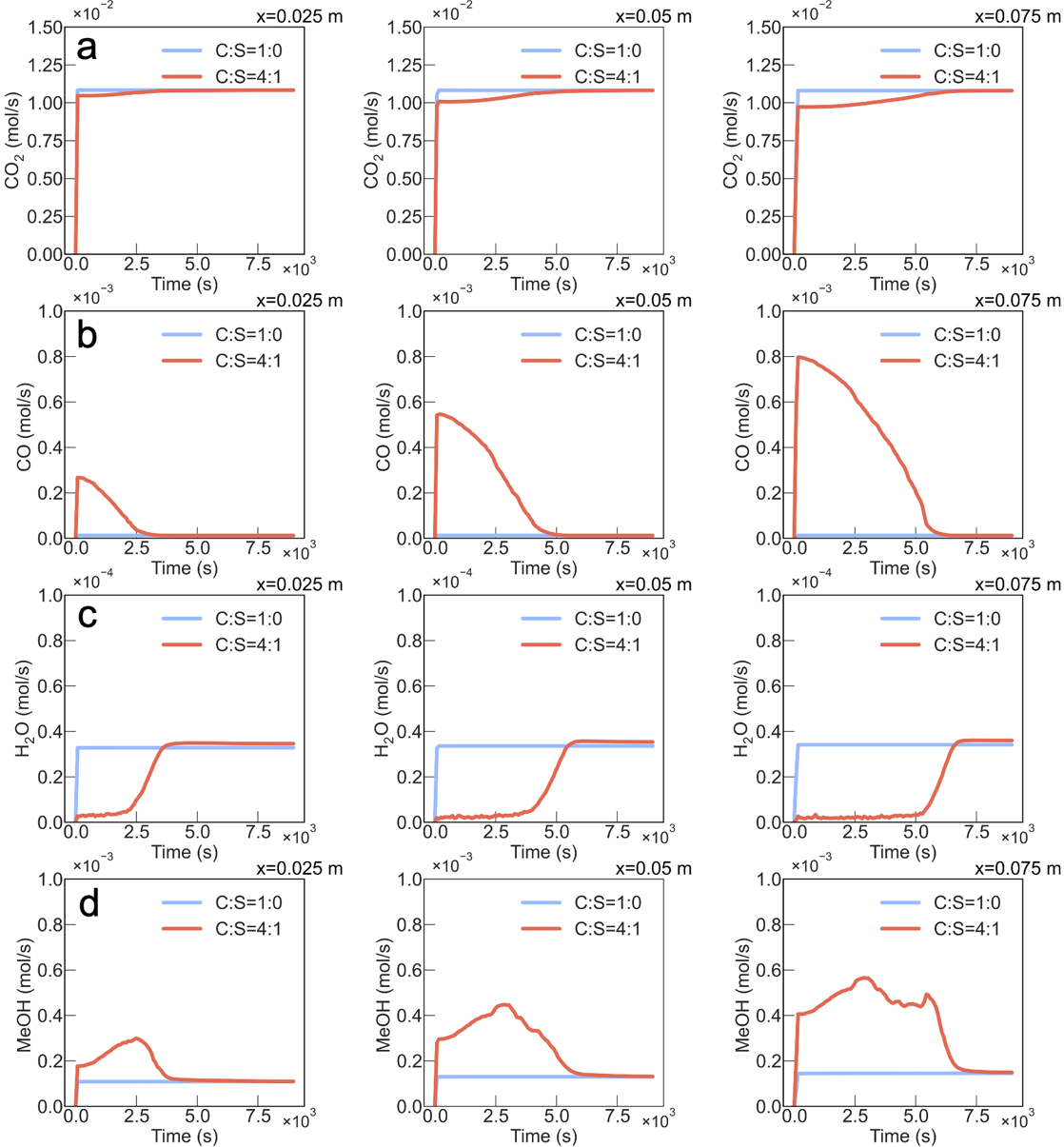
**

**Fig. S3.** Slice average rate of (a) CO_2_, (b) CO (c) H_2_O and (d) MeOH at different locations (along the flow direction) of the SER (C:S = 4:1) and catalyst-only (C:S = 1:0) systems.


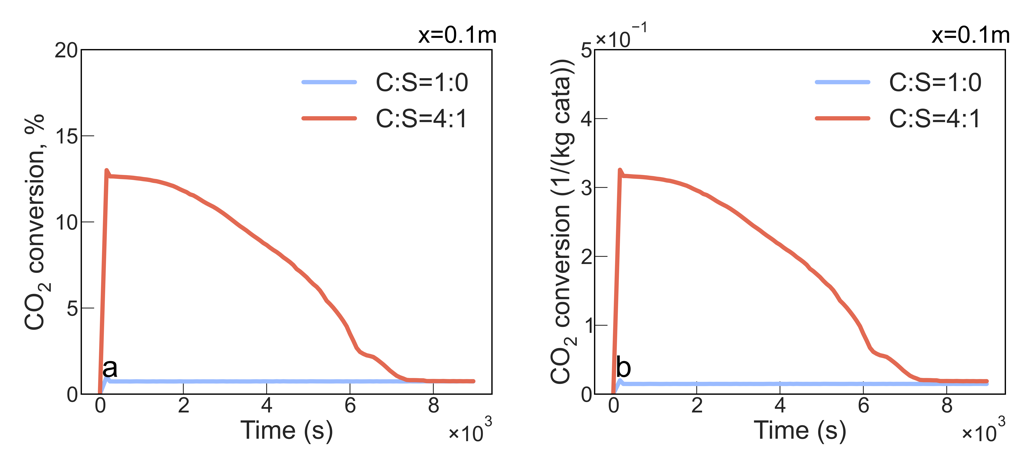


**Fig. S4.** CO_2_ conversion of the SER (C:S = 4:1) and catalyst-only (C:S = 1:0) systems: (a) simulated absolute conversion rate and (b) mass-specific conversion rate.


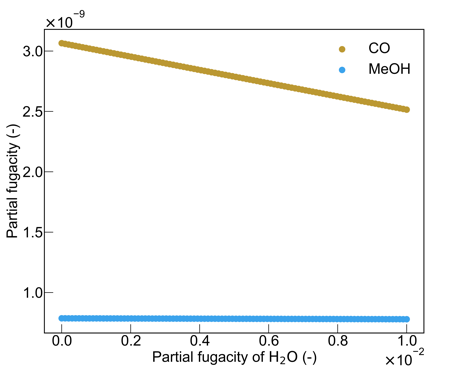


**Fig. S5.** Simulated CO and MeOH by the microkinetic model as a function of different H_2_O partial fugacity at 520 K, 5×10^6^ Pa, constant CO_2_ /H_2_ ratio at 10.


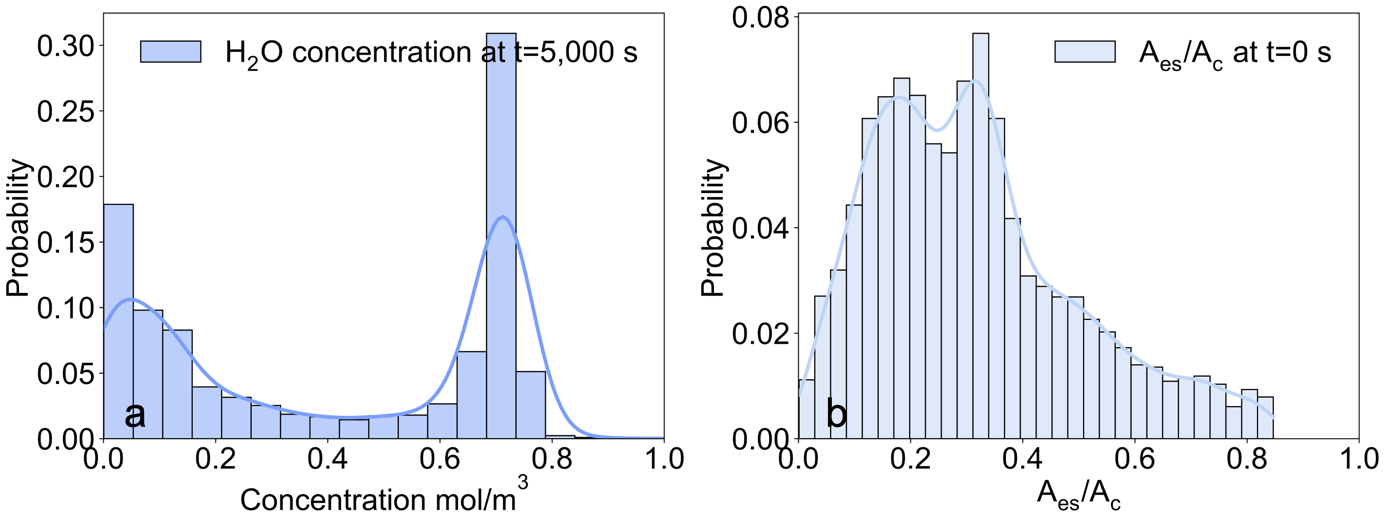


**Fig. S6.** (a) H_2_O concentration distribution within individual pores at t = 5,000 s;(b) A_es_/A_c_ distribution for individual pores at t = 0 s, where larger values indicate more sorbent coverage of the pore with stronger H_2_O removal capability.


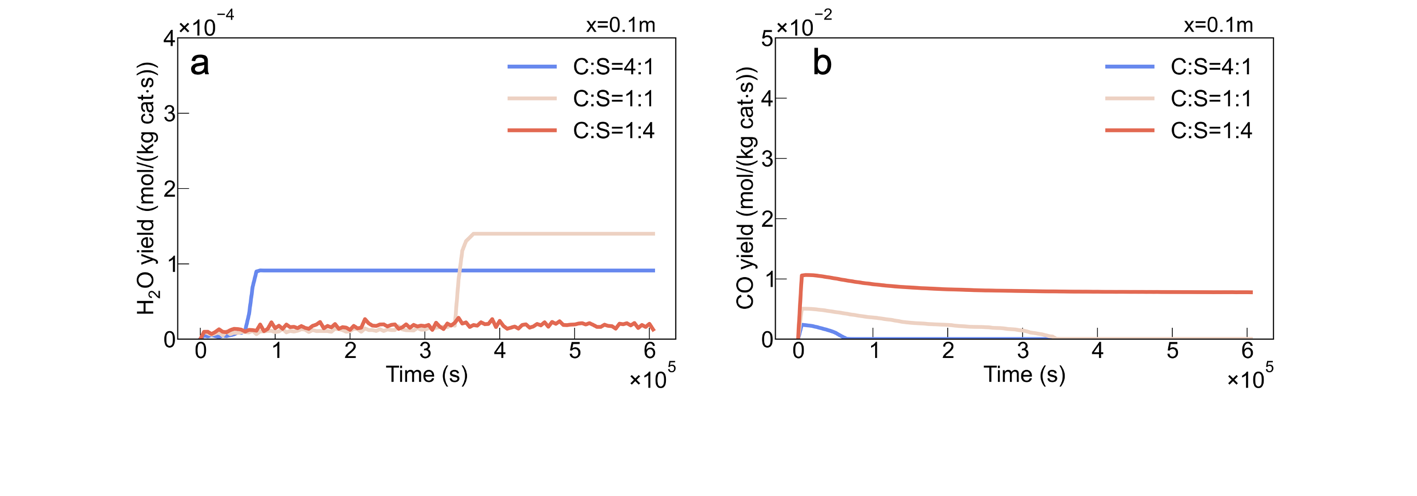


**Fig. S7.** Comparison of the catalyst mass specific product formation rate at the reactor outlet for: (a) H_2_O and (b) CO.

**Supplementary Note 4**

**Experimental demonstration that catalyst performance is enhanced with sorbents**

We performed laboratory experiments to validate the trends in reactor performance predicted by our numerical model. The experimental apparatus is shown in **Fig. S8**. The packed bed reactor had a height of 87 mm, with an outer diameter of 25 mm and an inner diameter of 11 mm. The inlet flow rate was maintained at 100 ml/min, and the reactor temperature was controlled at 513.15 K. The catalyst used was CuIn_0.5_/ZnZrO_x_ and the adsorbent used was 13X zeolite. The particle diameter varied from 0.2 to 0.5 mm. The reactor arrangement involved an inlet hydrocarbon ratio set at 1:10. Gas chromatography analysis was performed using an Agilent GC8860 to analyze the product at the reactor outlet at a 26-minute interval (14,000 s in total). The mass of the catalyst was consistently controlled at 3 g for each experiment, while the mass ratios of catalyst to sorbent were varied as 1:0 (catalyst-only), 4:1, 1:1 and 1:4. To ensure consistent packing mass (18 g) within the reactor, quartz sand of the same particle size was used in each experiment when varying the catalyst to sorbent ratio. The MeOH yield per unit mass of catalyst at the reactor outlet and the net increase in MeOH production rate per unit mass of catalyst ($\Delta N_{MeOH}/kg cat\cdot s$) are shown in providing insight into the impact of varying sorbent-to-catalyst ratios on reactor performance.


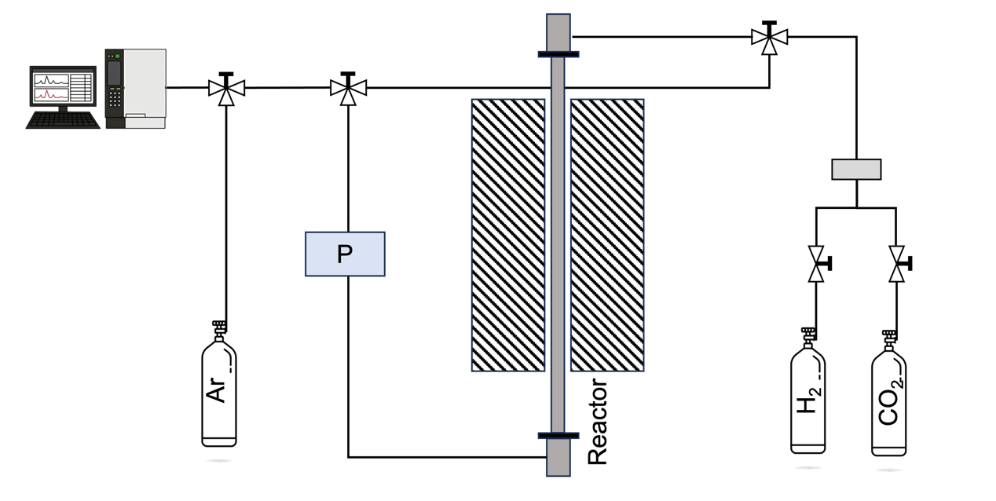


**Fig. S8.** Schematic of the experimental rig used in this study to perform catalytic CO_2_ hydrogenation over CuIn_0.5_/ZnZrO_x_ (with 13X zeolite).

**Fig. S9a** shows the CO₂ conversion rate with varying proportions of sorption particles. With the introduction of sorption particles, significant enhancement in CO₂ conversion was observed, with an increase of approximately 30%, 55%, and 70% in the peak conversion rate for C:S = 4:1, 1:1, and 1:4, respectively, **Fig. S9b** illustrates the corresponding CO yield. Similarly, **Fig. S9c** illustrates the corresponding MeOH yield, which also demonstrates an increase trend with increasing sorbent concentration.


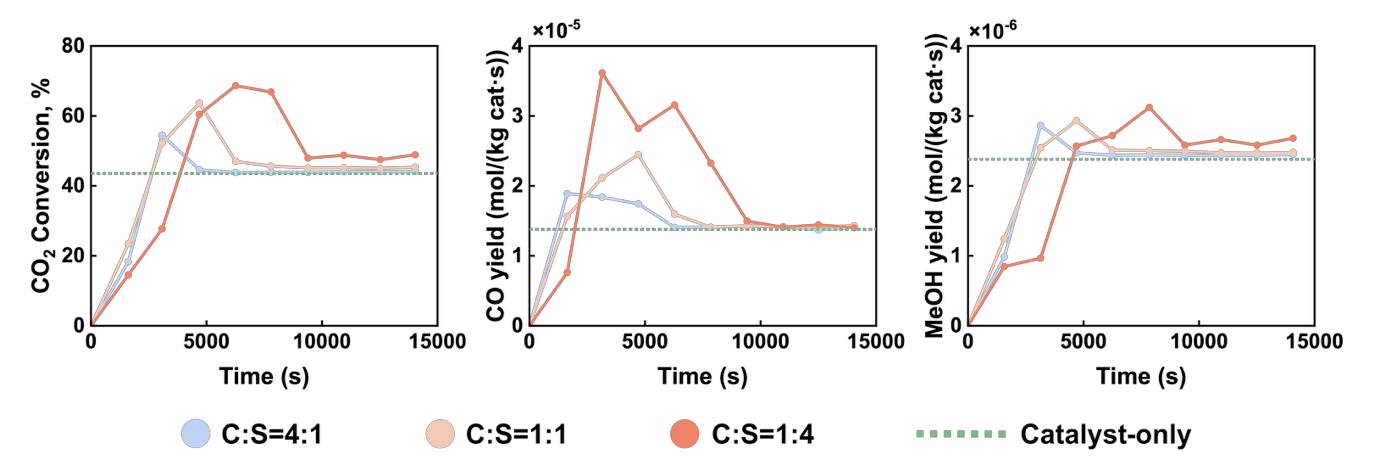


**Fig. S9.** The measured activity data (as a function of reaction time) for the SER systems (using and 13X zeolite) with different proportions of catalyst (CuIn_0.5_/ZnZrO_x_) and sorption (13X zeolite) particles: (a) CO₂ conversion rate, (b) mass-specific CO yield, and (c) mass-specific MeOH yield. The dashed line shows the experimental measurements.

**Supplementary references**

1. Graaf, G. H., Stamhuis, E. J. & Beenackers, A. A. C. M. Kinetics of low-pressure methanol synthesis. *Chem Eng Sci* 43, 3185–3195 (1988).

2. Vanden Bussche, K. M. & Froment, G. F. A steady-state kinetic model for methanol synthesis and the water gas shift reaction on a commercial Cu/ZnO/Al2O3 catalyst. *J Catal* 161, (1996).

3. Portha, J.-F. *et al.* Kinetics of Methanol Synthesis from Carbon Dioxide Hydrogenation over Copper–Zinc Oxide Catalysts. *Ind Eng Chem Res* 56, 13133–13145 (2017).

4. Bracconi, M. & Maestri, M. Training set design for machine learning techniques applied to the approximation of computationally intensive first-principles kinetic models. *Chemical Engineering Journal* 400, 125469 (2020).

5. Gao, S. *et al.* Performance of sorption thermal energy storage in zeolite bed reactors: Analytical solution and experiment. *J Energy Storage* 64, 107154 (2023).

6. Maksimov, P., Laari, A., Ruuskanen, V., Koiranen, T. & Ahola, J. Methanol synthesis through sorption enhanced carbon dioxide hydrogenation. *Chemical Engineering Journal* 418, 129290 (2021).
